# The importance of stakeholder selection in core outcome set development: how surveying different health professionals may influence outcome selection

**DOI:** 10.1186/1745-6215-16-S2-P47

**Published:** 2015-11-16

**Authors:** Kerry Avery, Katy Chalmers, Katie Whale, Natalie Blencowe, Rhiannon Macefield, Sara Brookes, Chris Metcalfe, Jane Blazeby

**Affiliations:** 1 University of Bristol, Bristol, UK

## Background

Core outcome sets (COS) seek to overcome heterogeneity in outcome reporting by generating outcomes to be measured and reported as a minimum in all trials of specific conditions. Patients’ views are integral to this but less is understood about selecting which healthcare professionals to be involved. We examined whether differences in the health professionals surveyed influenced outcome selection in a COS.

## Methods

Systematic literature reviews and patient interviews informed the development of a ‘long-list’ of outcomes. Outcomes were operationalised into items and sent to 96 healthcare professionals (73 doctors, 23 nurses) and 130 patients. Delphi consensus methods were used to prioritise items by asking participants to rate items from 1 (‘not important’) to 9 (‘extremely important’). Data from different professionals were compared with patients’ views. Results: 115 (88%) patients, 17 (74%) nurses and 52 (71%) doctors completed the 68-item questionnaire. Patients rated more items (53,78%) as essential than nurses (47,69%) and doctors (26,38%). Patients (23,61%) and nurses (31,80%) rated more patient-reported outcomes (PROs) as essential than doctors (8,21%). Whilst patients rated all adverse events as essential, only 12 (46%) and 15 (58%) were rated essential by nurses and doctors. The top three patient-rated items related to benefits of interventions, compared to nurses (PROs) and doctors (two adverse events, one benefit).

## Conclusions

Nurses have similar opinions to patients about incorporating PROs and benefits in a COS but have comparable views to doctors regarding adverse events. Every effort should be made to include key stakeholders such as nurses in COS development.

